# Ethical aspects of triage in civil and military contexts: a qualitative research of physicians’ attitudes and knowledge regarding ethics in triage scenarios in

**DOI:** 10.3389/fmed.2026.1938455

**Published:** 2026-10-20

**Authors:** Janna Seelig, Marcin Orzechowski, Florian Steger

**Affiliations:** Institute of the History, Philosophy and Ethics of Medicine, Ulm University, Ulm, Germany

**Keywords:** clinical ethics, mass casualty incident, military medicine, pandemic, triage

## Abstract

**Background:**

Triage in the medical context is a screening procedure used to determine resource distribution in situations of severe restriction of medical resources. Originally, triage was designed as a method to ascertain the treatment priority of wounded soldiers on battlefields. However, ethical discussions about clinical triage were raised during the COVID-19 pandemic. In the focus stood prioritization of care in situation of limited resources.

**Objective:**

The purpose of our investigation was comparison of experiences and ethical attitudes towards triage among military and civil physicians. To date, there is no research available on this topic.

**Methods:**

We conducted 26 semi-structured, qualitative, exploratory interviews among military and civilian physicians in Germany. All the interviews were digitally audio-recorded, transcribed and anonymized. We analyzed the transcripts via the structured qualitative content analysis method.

**Results:**

Triage scenarios bring distinctive ethical challenges for the attending physicians. Military and civil physicians share a similar approach to solve the ethical conflicts arousing in such situations. Triage situation does not allow for an individual medical gold standard in the treatment of patients; therefore, the principles of autonomy, beneficence and nonmaleficence should be subordinated to the principle of social justice. Because of restriction of the patient's autonomy, the final decision-making authority lies with medical professionals. Pre-clinical triage conditions also restrict patient-physician relationship.

**Conclusion:**

The ethical justification of triage follows the imperatives of life-preservation and maximization of the number of survivors. According to interviewed physicians, in both pre-clinical and clinical triage situations, medical decisions should aim rather at maximizing the well-being of the collective – whether of wounded or of a society – than at maximizing the well-being of an individual patient. Restriction of patient autonomy regarding treatment discontinuation – ex-post triage – is unavoidable in a pandemic situation.

## 1 Introduction

Triage in the medical context is a screening procedure used to determine resource distribution, especially in situations where medical resources for treatment are limited (1, 2). By recognizing the severity of an illness or injury in consideration of current available resources, the triage procedure aims to determine a priority for individual treatment. Originally, triage was designed as a method to ascertain the treatment priority of wounded soldiers on battlefields. The priority was the preservation of manpower and, secondarily, the health of the wounded soldiers (3, 4). Therefore, the purpose of triage in situations of existential emergencies is to generate the greatest possible benefit by using limited resources as efficiently as possible. To this day, in tactical medicine, care for individual soldiers must be subordinated to tactical circumstances if necessary. By accepting this prioritization in military scenarios, individual soldiers’ autonomy is highly restricted. This could stand in opposition to one of the ethical principles of biomedicine: individual autonomy of a patient (5), which is the central value in modern medicine that needs to be protected and respected (6).

In context of ethical deliberations about triage, it is important to differentiate between ex-post triage and ex-ante triage. Ex-post triage refers to the medical decision to discontinue intensive care treatment that has already begun for one patient in favor of a newly arriving patient with significantly better chances of survival. In such situation, for example, a patient already in the intensive care unit is disconnected from life-support equipment (such as a ventilator) so that the equipment can be reassigned to a newly arrived emergency patient. In case of ex-ante triage, on the other hand, a decision is made before treatment begins as to who will receive the last available resources.

Depending on the situation in which it is performed, triage can have a temporal dimension. Military triage on the battlefield or after a mass casualties incidents are typically relatively short and the number of potential patients can be roughly estimated. In a pandemic situation, the number patients who require triage is difficult to assess as well as the duration of the situation, during which triage will be necessary. This can put an enormous strain on the medical resources assigned to care for patients.

Medico-ethical and social discussions about triage intensified in many countries, including Germany, during the COVID-19 pandemic (6–8). The focus of these debates was on how physicians should decide, who should receive intensive care when resources such as intensive care unit (ICU) beds or ventilators are insufficient. Key points of the debate concentrated on whether decisions should be based on medical criteria such as the likelihood of survival or rather on factors such as age, disability, or social status. Disability advocacy groups criticized that purely utilitarian approaches could disadvantage people with disabilities (9, 10).

Although the four bioethical principles constitute fundamental ethical considerations of a patient's treatment and are accepted as the central professional ethic conventions for physicians, there are differences in how physicians evaluate and implement norms related to resource allocation in various triage scenarios, especially in comparison of military and nonmilitary triage. To date, there is no research available on the experiences and ethical attitudes of military and civil physicians towards triage (6). From a medico-ethical perspective, it is of interest to understand the ethical argumentation behind prioritization in military scenarios to learn from it for civil triage scenarios, such as pandemic or mass casualty incidents. Therefore, our research aimed to explore applied ethical norms and physicians’ decision-making processes in military and nonmilitary triage scenarios. The central research questions for our research were as follows: (i) What are the different characteristics of the following three triage scenarios: pandemic, mass casualty, and military setting from the military and civil physicians’ point of view? (ii) On which ethical principles should triage decisions be oriented? (iii) Is there a difference in the ethical evaluation of triage scenarios between military and civilian physicians?

## 2 Materials and methods

Since making decisions about prioritizing patients’ treatments is a medical task for physicians, we conducted 26 semi-structured, qualitative, exploratory interviews among physicians in Germany to assess their different ethical perceptions of triage. This method of scientific research does not aim at generalizing the results but is suitable for collecting the subjective views of the interviewees, while considering their individual background and experiences. In addition, the method of qualitative interviews allows for certain flexibility in the interview process through the possibility of focusing on certain specific issues mentioned by the interview partners, asking additional questions, and clarifying statements of the interlocutors (11).

To access possible interview partners, a purposive sampling design was used (12, 13). The recruitment process was conducted in hospitals with departments of Anesthesiology, Surgery and Internal Medicine. To recruit interviewees with military backgrounds, we contacted military physicians in all five German armed forces military hospitals (Bundeswehrkrankenhäusern). Additionally, we followed some of the recommendations given by military physicians, who referred colleagues working in specialized units in the German Armed Forces. For recruitment of the group of civilian physicians, we contacted civil physicians in maximum provider hospitals, trauma centers and regional hospitals. These hospitals were distributed in various cities Germany-wide. The choice of the level of care and location of the hospitals was motivated on two grounds. First, the variation in the level of care and choice of different military units was intended to provide us with information about the different experiences of physicians since the scarcity of resources differs across the levels of care. Second, the locations of hospitals depict the representation of the physician population in Germany. The chosen hospitals were located in various German federal states, since the federal states were to varying degrees affected by the COVID-19 pandemic.

The potential interviewees were contacted via email. Participants were recruited through the chief physicians’ offices and the respective departments. In this initial communication, we introduced our project and asked for participation. The criteria for participating as an interviewee were (i) working as a physician in one of the specialties mentioned above AND (ii) working as a physician during the COVID-19 pandemic AND (iii) working in a civil clinic OR (iv) working as a military physician in the German Armed Forces (Bundeswehr). The response rate from the chief physicians’ offices was 67%. We aimed for a balanced gender ratio and a balanced ratio between civil and military physicians. In the interviews took part 14 military and 12 civilian physicians; we have interviewed 11 female and 15 male interlocutors. Due to qualitative nature of the investigation and because the research questions do not specifically study the influence of individual characteristics of interviewees, i.e., age, career path or professional background, on the research topic, no demographic data were collected during the interviews.

All 26 interviews were conducted personally on site or alternatively by telephone or via a secured digital telecommunication platform. The interview guide consisted of 13 questions based on the ethical principles of Beauchamp and Childress (5), which were complemented by *ad hoc* questions to clarify the respondents’ statements. Before the interviews, all interlocutors were informed about the measures to safeguard their anonymity. Moreover, the interviewees were informed about the aim and conduct of the research and that their participation in the project was voluntary and could be revoked at any time. The interviews were conducted only after the interviewees consented to participate in the research. The interviewees took part in the research on a voluntary basis and were not compensated in any way for their participation.

The interview questions were developed for the purpose of the study and have not been published before. All the interviews were digitally audio-recorded, transcribed and anonymized. We analysed the transcripts via the structured qualitative content analysis method described by Mayring (14). First, the content of the interviews was reduced to core elements. Second, the extracted elements were systemized into main topics and subtopics. For the coding process, we used the software program MAXQDA to ensure a consistent and structured analysis of the extracted elements. The interviews, as well as the processes of transcription, coding, analysis, and interpretation, were conducted personally by the project researchers.

Before the beginning of the research, the study protocol was submitted to the local Institutional Review Board. Ethical review and approval was waived by the board, as no information on personal data, personal data of patients, health-related data or data on sexuality of the interviewees was collected.

## 3 Results

Our results focus on ethical challenges and solutions for applying triage in different scenarios, such as pandemic, mass casualty and military settings. Therefore, we first present the similarities and differences among the given triage scenarios. Additionally, we present an analysis of the different triage scenarios following the ethical principles of biomedicine.

### 3.1 Characteristics of the various triage scenarios: pandemic, mass casualty, and military setting

Our interlocutors pointed out to different characteristics of the three triage scenarios (Table 1).

**Table 1:** Different characteristics of the three triage scenarios (military, mass casualty incident, pandemics) pointed out by the interviewees.

	**Triage Scenarios**		
	**Pre-clinical**		**Clinical**
**Characteristics of Triage**	Military Scenarios	Mass Casualties (MASCAL)	Pandemics
**Aim/Purpose**	Rescue	Rescue	Therapy
**Course**	Short-time overload of rescue rescources in a limited system	Short-time overload of rescue resources embedded in a well structured healthcare system	Short, mid and longterm overload of the healthcare system
**Dynamics**	Dynamic or fixed after event X due to the development of the tactical situation	Fixed after event X that led to an MASCAL, but dynamic development due to a second hit possible	Dynamic due to an increasing number of patients and causes of the pandemic
**Place**	Combat scenarios in less developed infrastructures, high risk setting	Highly developed infrastructure, setting`s risk depends on the cause, shifts to protected setting (clinics, hospitals)	Highly developed infrastructure, protected setting (clinics, hospitals)
**Patterns of injury**	Ballistic trauma, amputations, critical bleeding etc.	Trauma, critical bleeding	Infectious diseases
**Group of patients**	Soldiers: younger and more homogenous group regarding health status than the general public, comraderie as an emotional burden	Variable age and health status of patients	Variable age and health status of patients
**Resources**	Highly limited material, personal and structural resources	Limited resources at the scene, but adequate availability of material and personal resources in the protected setting, high availability of structural resources for transportation and clinical supply	Throughout the pandemic`s progress incriesingly high limitation of personal, structural and material resources, special feature: demand of a certain material resource
**Decision-making**	Algorithm based	Algorithm based	No algorithm, no national standard

All the military physicians emphasized the priority of self-protection in combat settings. The respect for the tactical circumstances is always above every medical action. Combat action influences the extent of medical measures directly by tying up personal and material resources. Furthermore, the interviewed military physicians stated that the infrastructure in military settings was a challenge for planning the transport of the wounded. Additionally, the wounded soldiers themselves represented a special feature of medical care in military triage scenarios. Since camaraderie was described by the interviewed military physicians as a unique personal bond between soldiers, it could lead to emotional distress for the attending physicians.

In a military setting, where you have someone in front of you in the same uniform or allied uniform with whom you previously ate together in the care facility, this is certainly a different stress than in a mass casualty incident on the motorway, where you may, no, certainly, do not know any of the injured. (military physician 6)

The interviewed military physicians, as well as most civil physicians, noted the variety of mass casualties resulting from traffic accidents, natural disasters, and terrorist attacks (MASCAL). This led to the assessment of the surveyed physicians that mass casualties were unique and rare events that could hardly be generalized. Every mass casualty triage scenario has its own characteristics with respect to its cause, number of injuries, injury patterns and threat. Most of the military physicians compared a mass casualty triage scenario caused by a shooting or terror attack to triage scenarios in military settings. Both situations were tactical scenarios with security considerations, in which self-protection and respect for the tactical circumstances were prioritized. The interviewed military and civil physicians all stated that triage never should follow social criteria instead of medical and objective criteria.

I see triage as a tool to get out of the chaos phase. When it works well, in such situations, whether civilian or military, in emergency medicine or infectious disease scenarios, there is always a phase of chaos, and triage is designed to help move out of that phase as quickly as possible. (military physician 9)

These [refers to mass casualty and military scenarios] are often isolated or individual events, and one situation cannot be compared to another. This makes it very difficult to speak about such cases in general terms. It is clear that the factor of time always plays a role. Concepts such as justice and equality must also be considered within the context of time. (military physician 8)

The interviewed physicians noted that a pandemic not only affects the medical sector but also has political, economic, and social impacts. Therefore, during the pandemic, the priority shifts from individual infection treatment to collective pandemic management. First, the prolonged temporal approach was identified as a challenge in pandemic triage scenarios. A growing patient base over time was considered the main reason for the overwhelming clinical capacity. Since casual treatment for COVID-19 did not exist, patients stayed in hospitals long-term. Exit-blocks and overcrowding were the main factors that physicians had to deal with during the pandemic because they led to a prolonged and more excessive need for limited resources. Second, triage during a pandemic does not pursue the goal of rescuing patients by prioritizing them in short-term emergency scenarios. Instead, medical clinical treatment should be prioritized on the basis of a medical indication. Therefore, for some physicians, triage in the context of a pandemic was a maximum enhanced version of the everyday allocation of scarce resources.

The pandemic is unique in that its duration is significantly longer than that of a mass casualty event. In a mass casualty event, there is a certain influx of patients of quantity X, after which there are no more patients from that scenario. During the pandemic, however, there is a continuously emerging group of patients who will have claims on resources in the future. This is something I constantly have to keep in mind and consider. However, this does not mean that resources should be withheld. This means that there is increased competition for these resources. (civilian physician 2)

### 3.2 Ethical assessment of various triage situations

The surveyed physicians clearly differentiated between pre-clinical and clinical triage scenarios based on their characteristics and special features. While pre-clinical triage should concentrate on screening, clinical triage focuses on prioritization of treatment. Although there are similarities in ethical assessment of both scenarios, visible in the interviews were differences highlighted by our interlocutors. These differences involved issues of autonomy of patients, beneficence – especially affected by patient-physician relationship – and social justice (Table 2).

**Table 2:** Similarities and differences in pre-clinical and clinical triage scenarios from the perspective of four ethical principles pointed out by the interviewed physicians.

	**Pre-clinical triage scenarios (military triage, mass casualties)**	**Clinical triage scenario (pandemic)**
**Autonomy**	severely restricted difficult or impossible to consider active refusal of medical measures as exclusive expression of autonomy	less restricted depending on patient's condition and resource situation determination of patient's will through living will or communication with relatives
**Beneficence**	depending on the available resources, no maximum therapy but only the best possible treatment in the respective situation patient-physician relationship: communication limited or impossible building of trust impossible or severely limited	depending on the available resources, no maximum therapy but only the best possible treatment in the respective situation patient-physician relationship: communication with patient or relatives possible relationship strongly based on gradual build-up of trust
**Nonmaleficence**	acceptance of individual harm in order to generate as much benefit as possible for the collective of wounded only limited treatment to save resources	acceptance of individual harm in order to generate as much benefit as possible for the society and to contain the pandemic
**Social justice**	prioritization based only on current condition of patients rejection of the decision based on social criteria treatment prioritization based only on objective medical parameters ex-post triage ethically allowed	prioritization based on medical history of patients rejection of the decision based on social criteria treatment prioritization based only on objective medical parameters ex-post triage ethically allowed

#### 3.2.1 Autonomy

The ethical principle of respect for autonomy recognizes individuals’ right to make self-determined decisions about their own lives and health. This requires that patients are fully informed to enable them to give informed consent. The interviewed physicians emphasized respect for patients’ autonomy in modern medicine. However, most of them stated that patients’ autonomy is highly limited in triage scenarios. Especially in preclinical triage settings, most military and civil physicians mentioned that it was highly unlikely or impossible to respect patients’ autonomy in the context of informed consent. In these scenarios, it is the physicianś responsibility to decide in the best interest of patients. The interviewed physicians assumed a general patient´s will to survive and to be cared for, which justified the limitation of the patient´s autonomy towards the decision-making power of physicians. The scarcer the resources were, the more limited the patient´s autonomy was.

Honestly, if we perform triage properly, the only factor that determines the decision is whether someone has a better chance of survival or not, which is decided upon by the physician. (military physician 14)

No, patients’ autonomy does not exist at all. I believe triage is precisely the kind of scenario where individualized medicine is no longer possible, and every patient has, more or less, relinquished their autonomy. (civil physician 11)

#### 3.2.2 Beneficence

The ethical principle of beneficence is the obligation to actively contribute to patients’ well-being and to take actions that promote their health and welfare. This includes balancing actions to maximize benefits while minimizing harm. The interviewed physicians concluded that triage decisions, regardless of the scenario, would be the most challenging decisions physicians could make. From the perspective of interviewed physicians, the extent of the benefit to individual patients is limited in triage situations, which is why physicians bear the responsibility for decisions regarding resource allocation. This responsibility is exacerbated by the fact that patients have to give up some of their autonomy in triage situations.

The interviewees pointed out that triage situations influence the patient-physician relationship. In such situations, as patients largely give up their autonomy and physicians take on additional responsibility, decisions need to be made more rational and pragmatic. According to interviewees, the patient-physician relationship significantly relies on trust. Patients rely on physicians deciding professionally, not on the basis of personal likes or dislikes. The military and civil physicians mentioned that honest and open communication with patients or relatives was crucial for strengthening trust in the relationship. The prognosis should be established on the basis of medical criteria without giving the impression that resources are redistributed in favour of other patients. Some physicians mentioned that patients in clinical triage scenarios might fear being deprioritized or disadvantaged, which could strain the patient-physician relationship.

Since triage decisions are about life and death, physicians carry an extremely high duty of care and diligence in this case. It must not be done negligently but thoroughly. That means you cannot help every patient, but you are trying to help most of them. As a physician, that means that the relationship with my patients is highly strained. (military physician 2)

Triage decisions are the hardest to make in modern medicine. Physicians carry the burden of responsibility not only for the individual patient but also for the collective of patients. That sometimes means that you have to make paternalistic decisions and cannot keep up with the standard of informed consent. That is something you can afford in a life existential crisis. However, that does not mean that you want to harm a patient; deciding more paternistically and pragmatically in triage scenarios is a mechanism to decide more efficiently, which rescues more patients, generating greater benefits. (civil physician 1)

#### 3.2.3 Nonmaleficence

The ethical principle of nonmaleficence includes the obligation to avoid causing harm or suffering. Medical actions should be designed to avoid unnecessary harm, with existing risks minimized. The physicians indicated that a fundamental principle of triage was that harm had to be accepted. The interviewees viewed triage as a method to manage the resulting harm. From their point of view, individual harm would have to be accepted in order to generate as much benefit as possible overall in the patient collective. Both the material limitation of resources and the exhaustion of personnel resources could limit the effectiveness of therapy for individual patients. Therefore, it would not be possible to provide maximum therapy but only the best possible care given the circumstances. The fewer resources available, the greater the physicians’ responsibility to make decisions not only for individual patients but also for the patient collective. Many physicians referred to it with the words: “do the most for the most part”.

This is exactly the issue; there is a maximum imbalance, which is justified by the principle of justice. The balance between beneficence and harm for the individual patient. Typically, we do not accept this type of imbalance. This is especially the case since we themselves are the ones who cause imbalance. Typically, we stop therapy in the event of an imbalance, adjust it, and change the therapeutic goal. When the scales tip on their own, when we simply see that our treatment is not working, or that the side effects outweigh the benefits, whatever it may be, that's the point at which we always say, independent of the autonomy perspective, this is the moment where we say, we no longer have a therapeutic goal, meaning we no longer have a curative treatment option. In triage, the horse is essentially put before the cart. Justice makes it necessary for us to take the balance beam into our hands and lift it. I believe this is the major issue with the triage decision. (civil physician 8)

You can only do the most for the majority, but you cannot expect to do the best for each individual. That's simply not possible. (civil physician 10)

I believe this is also why the term triage fits so well in the context of the pandemic. After all, it originally came from the emergency medical and military context, where the focus was more on collective success rather than individual treatment. (military physician 9)

The physicians indicated that considerations for conducting ex-post triage arose. All the surveyed military and civil physicians considered ex-post triage to be an important feasible measure if the resource situation demanded it. The military and civil physicians emphasized that ex-post triage is not about weighing survival probabilities between two patients competing for an intensive care bed but rather about an individual medical decision. Each patient must be individually assessed on the basis of medical criteria to determine their prospects of surviving the current infection through therapy. Both military and civil physicians stressed that the course of therapy must be constantly re-evaluated since the progression of the individual disease, resource availability, and patient intake are dynamic.

Especially when the situation continues to escalate, I no longer have sufficient capacity to do justice to all patients. This is precisely where the core of triage lies. The question should not be whether I discontinue treatment in favour of another patient but rather whether it is justifiable to accept the harm caused to an individual by discontinuing treatment owing to a lack of resources. In my opinion, this would be justified, but only in an absolute emergency or exceptional situation. For example, the pandemic would have been. (military physician 1)

I believe this is feasible, but it requires a well-structured, written process. Because simply determining that someone no longer has a chance sounds straightforward, it requires extensive expertise, potentially involving multiple specialists, especially concerning interdisciplinary cases and injury patterns. (civil physician 5)

#### 3.2.4 Social justice

The ethical principle of social justice claims a fair and equitable distribution of resources and services without discrimination. It involves ensuring equal access to medical care for all people, regardless of social, economic, or other factors. The term refers to distribution of healthcare resources to individuals within larger groups of population, for example to individual patients during a pandemic situation or to individual casualties on the battlefield or after a mass casualties incident.

Military physicians, as well as several civil physicians, considered medicine and the healthcare system in general to be unjust and based on inequality and a lack of resources. It could not be the task of doctors to make an unjust system just. However, it was their function to act to the best of their knowledge and assessment, to ensure equal standards among patients and to avoid making arbitrary decisions. Some physicians have described ubiquitous inequality in medicine on the basis of biological and genetic individuality. This inequality pertained to the individuality of patients and should be accepted and not changed. Given that individual patients are still considered in triage situations and have different preexisting comorbidities, it is necessary to assess individually the success prospects of therapy or measures in each acute situation. Therefore, it was logically derived by the interviewed physicians that unequal patients would be treated unequally. This inequality leads to different chances for patients.

The mistake in the discussion about triage is to assume that patients are the same. We do not have that. Every individual patient needs their individual prognosis. (civil physician 2)

It is indeed the case that, for most of us, justice means giving priority to those who have a fair chance of surviving the situation over those who, on the basis of everything we can medically assess, are unlikely to benefit from treatment. I believe this is a different perspective and a different sense of justice than some politicians might have. (military physician 7)

According to the interviewees, focusing on equality in clinical practice would inevitably lead to a random first-come first-served principle or lottery procedure detached from medical standards. Both the military and civil physicians found such triage concepts unprofessional and unacceptable since they would be notably less fair for the collective than conducting triage.

If I do not use triage, it is the most unfair scenario because it means that you make decisions on the basis of a lottery. In addition, that is completely detached from medical knowledge and standards. (civil physician 9)

#### 3.2.5 Summary of results

The results of the interviews show that triage scenarios bring distinctive ethical challenges for the attending physicians. First, characteristic is emphasis on which ethical principles decisions during triage should be made. Since, according to the interviewees, a triage situation does not allow for an individual medical gold standard in the treatment of patients, only the best possible approach can be taken in such situations. The interviewed physicians saw the focus on the best possible solution as a reversal of the ethical evaluation of individual medical decisions. The principles of patient autonomy, beneficence and nonmaleficence should be subordinated to the principle of social justice. The guiding principles “do the most for the most” or “the greatest good for the greatest number” were mentioned by many of the interviewees as central approaches in triage scenarios.

Second issue that became distinctive during the interviews is the question of decision-makers. While in everyday clinical practice, the final decision-making authority lies with the patient, the surveyed physicians uniformly stated that in triage scenarios it ultimately lies with medical professionals. It is the doctor's task to decide which therapeutic measures are indicated for individual patients; and this decision is based on the availability of resources, the number of patients requiring help and the individual prospects of success. In a triage scenario, the individual patient is therefore unable to make an autonomous decision, as they generally lack knowledge of the whole triage situation. However, patients would still have the right to refuse measures.

Third, the ethical evaluation of triage scenarios did not differ between military and civil physicians. As the results above have shown, military and civil physicians share an approach to solve the ethical conflict that accrues in triage scenarios. The principle of social justice substantiates accepting harm on an individual level to create the greatest possible benefit on a collective level. This utilitarian approach seems to be the only ethically acceptable evaluation of triage scenarios for the majority of interviewed physicians.

## 4 Discussion

We proceed by discussing central ethical challenges on the basis of the responses of our interviewees. As the presented results show, according to our interlocutors, the necessity of conducting triage should always be seen as the last resort. Only when the lack of resources cannot be remedied despite the optimal use of local and surrounding capacities, it must be decided which patients should be treated first. In a scarcity situation, a distribution procedure must be found that minimizes the negative consequences of triage; however, at the same time, it maintains the ability to act.

### 4.1 Insight into military medicine: acceptance of a silver standard

In military triage situations, it is assumed that the exhaustion of resources inevitably leads to the medical gold standard being unsustainable and drastically declining. The medical gold standard refers to the method, procedure, or technology in medicine that is considered the current best and most reliable option for diagnosing and treating a disease. In military triage situations, a so-called silver standard should be accepted.

This stricter utilitarian approach to care during times of limited resources focuses on the aggregate good of the group and gives no weight to the needs of individual patients (15). This representation can be interpreted as the subordination of patient's autonomy to social justice. Under a silver standard, patient-oriented, individual decisions should still be made but not aimed at maximizing the well-being of individuals but rather at maximizing the well-being of the collective. However, this stricter approach misses the ethical aspect of care for patients, who are expected to die based on the severity of their injuries and availability of resources. Inclusion of “end-of-life” care for dying patients is viewed as crucial for military triage situations (16).

The silver standard interpretation aligns with the ethical arguments of the interviewed physicians. The interviews revealed fundamental differences in the characteristics and challenges between pre-clinical and clinical triage scenarios. Pre-clinical triage methodology was described by the interviewed physicians as an as existential screening, and in-hospital triage was described as priority-oriented work. Despite these different approaches, no differences were found in the ethical argumentation of the both groups of interviewed physicians.

The literature questions the transfer of the utilitarian approach of military triage to civil scenarios. Although contemporary approach to military triage foresees – from ethical and humanitarian standpoint – that no distinction should be made between allied, enemy, or civilian casualties, historically the objective of military triage was to maintain defence capability of own forces (4, 17). In this context, soldiers find themselves in a sacrificial situation to achieve an external goal. Brettel et al. evaluated this traditional attitude as incomparable to clinical scenarios where triage decisions affect the immediate self-interests of patients (17).

However, the results of the interviews may indicate a contrary view. The arguments provided by our interlocutors were based on the initial principle that combating a pandemic takes precedence over treating individual COVID-19 infections. Therefore, the focus on combatting a pandemic can be understood as an external and preeminent goal. In the ethical reasoning of physicians, a situation emerged that presumed a collective objective, justifying the prioritization of the pandemic situation. The return to the gold standard could be achieved as quickly as possible for all patients. On an immediate individual level, only the silver standard could apply in an acute resource scarcity situation, relegating the individual interests of patients to a secondary position. Hence, civil patients in a clinical triage scenario would also be subjected to an external goal of benefit for the collective – similarly as in pre-clinical situations (18).

The interviewed physicians prioritized their triage decisions according to the principle of social justice. They argued that a need-oriented decision is efficient if it is made on the basis of uniform medical criteria and thus it is fair. This decision does not have to be fair to the individual but rather to the collective. From the argumentation in the interviews emerged an image of a collective patient that replaces an individual patient. This collective will is based primarily on the imperatives of maximization and life-preservation. The maximization imperative aims at prioritizing patients who are likely to gain significant health benefits from resource expenditure at the individual level. On a collective level, the maximization imperative is interpreted as maximizing the number of survivors. The life-preservation imperative prioritizes patients on the basis of medical urgency, aiming to prioritize those facing immediate significant health threats (19, 20). The arguments of the interviewed physicians suggest that a mixed approach of maximization and life-preservation imperatives is used in both preclinical and clinical triage scenarios. Depending on the extent of the scarcity, patients with the highest need, lowest resource expenditure, and highest chance of success are prioritized. This approach of utilitarian interpretation of triage was consistently observed among the interviewed military and civil physicians.

The literature suggests that a utilitarian orientation of triage in pre-clinical scenarios is ethically less problematic than in clinical scenarios (21, 22). This is referred to as ex ante consensus (23). Clinical triage, however, is discussed much more extensively in the literature. Interestingly, no ex post consensus, such as the ex ante consensus of pre-clinical triage scenarios, can be found. In civil and military emergency and rescue medicine, screening algorithms based on medical parameters are used (1, 24). These were referred to by our interviewed physicians as “hard facts”. However, such “hard facts” did not exist at the time for classification of COVID-19 infection and it is doubtful that they will be available in case of a future pandemic. Therefore, clinical prioritization decisions should be based on clinical prognosis (24). Interestingly, in the literature, orientation on the basis of clinical prognosis is viewed as a competitive situation, creating a prognosis comparative to other patients (19, 25). Such a competitive relationship between patients means that the allocation of survival probabilities depends on medical condition of other patients. Thus, survival probabilities should be considered relative, not absolute. Interpreting the interviewed physicians’ statements in this context, the competitive relationship is not seen as an influencing factor on the distinction between pre-clinical and clinical triage scenarios. According to the reasoning of our interviewees, one could go so far as to say that it makes no difference at all to the ethical assessment of a triage decision whether the patients are in a competitive relationship due to an absolute or relative shortage.

### 4.2 Ex-post triage as an ultimate form of comparative prioritization

Ex-post triage is considered the ultimate form of comparative success prospects. However, Patient A never competes only with Patient B, and all patients requiring intensive care are always considered. The German government banned ex-post triage in 2022 (26–28). However, on 23 September 2025, the Federal Constitutional Court of Germany declared the entire federal triage provision void. The Court did not rule that ex post triage is constitutionally required or permitted. Instead, it held that the federal government lacked legislative competence to regulate triage. If triage is to be regulated legislatively, it must generally be done by the German states rather than by the federal legislature (29). The following section focuses less on the legal assessment of ex-post triage and more on the ethical assessment of the ex-post triage dilemma.

Contrary to legal regulations and criticism – the interviews were conducted before the rule of the Federal Constitutional Court – all interviewed physicians advocated for the implementation of ex-post triage in absolute allocation crises. The implementation of ex post triage, such as an orientation towards clinical success prospects, is a medical measure based on scientific findings (15). This basic assumption was shared by most physicians participating in the interviews. Critics of ex post triage argue that discontinuing therapy for patients in favour of others through a forced change in treatment goals is not permissible, as it may contradict the patients’ wishes (25, 30). The surveyed physicians considered the restriction of patient's autonomy regarding treatment discontinuation unavoidable. Since it is only possible to do the best depending on resources and situational developments, the medical duty of care is particularly notable here. The unavoidability of ex-post triage can be interpreted as part of accepting the silver standard of care in triage situations. According to the postulate of equity contained in the ethical principle of social justice, incoming patients must be assessed on the basis of the same medical knowledge. If only admitted patients are treated and thus prioritized, this would automatically disadvantage all future patients in need. Therefore, rejecting ex-post triage can be interpreted as a “first-come, first-served” situation. However, the medical duty of care is not limited to already treated patients but also applies to those in need who come later. From the perspective of our interviewees, triage via a lottery or “first come first served” principle are medically unacceptable. Supporting arguments for this can be found in the literature. Orientation on the basis of the “first-come first-served principle” is considered ignoring scientific knowledge, resulting in a passive and self-selecting resource allocation that worsens outcomes. Adhering to the “first-come first-served” principle would mean that starting therapy establishes a permanent entitlement that excludes persons affected by future emergencies from appropriate consideration (17, 28).

In Germany, with the entry into force of the so-called Triage Act, on December 14, 2022, the explicit forbiddance of ex-post triage was decided by the German Parliament. This provided a decision; however, did not conclude a lengthy debate on the topic. The main argument for the prohibition was protection of the medical and legal situation of patients whose treatment already begun. In other words, if ex-post triage was permitted, a treated patient would have to permanently fear for his or her future care. However, this prohibition of ex-post triage is critically viewed by many physicians, lawyers, and ethicists. They mainly argue that it leads to the “first-come first-served” situation – precisely as the physicians, who were interviewed in our investigation. The decision by the Federal Constitutional Court declaring the federal triage provision void reopened this debate.

### 4.3 Limitations

To assess the results of this research properly, its limitations need to be considered. The first concerns the generalization of the results. The cohort of participants does not allow for generalization from a global perspective. However, the goal of qualitative interviews in research on questions of applied ethics lies in the presentation of subjective views of the participants. The data saturation achieved with the sample was sufficient to answer the research questions. On the other hand, the size of the sample provides an advantage — it allows a detailed assessment and comparison of interviewees’ statements. Future research could focus on surveys with higher number of participants from various countries, which would improve generalization of results and comparison in international perspective. Despite the satisfactory response rate, response bias should also be considered a limitation of the research, as it is expected that physicians willing to participate in the interviews were primarily those interested in the topic or had an experience with triage scenarios. Furthermore, the data collection method involving personal, telephone, or video interviews could influence the extent of physicians’ responsiveness. Moreover, it should be noted that the results of this research are not universally applicable. The original conception of triage in the military and its current application in civil medicine have been scarcely compared in research between civil and military physicians, rendering the derived questions exploratory in its nature. In addition, during the COVID-19 pandemic, there were no clinical triages in Germany involving treatment discontinuations or ex-post triage scenarios. Therefore, all assessments by surveyed physicians applying to such a situation should be considered theoretical and not based on practical experience in this aspect.

## 5 Conclusion

The present research addresses the question of the ethical justification of triage decisions in both military and civil contexts. Using a qualitative research approach, conducted were expert interviews with military and civil physicians. The aim of the investigation was to ascertain, which ethical principles are taken under consideration by physicians in triage scenarios, which principles are emphasized, and whether the ethical evaluation of military physicians differs from their civilian counterparts. Given the limited evidence on these questions in the literature on the topic up to date, the investigation aimed to fill out an important research gap. To our knowledge, this is first such empirical investigation with consideration of this research question.

The results show that there are no substantial differences in ethical assessment of the triage between military and civil physicians. Both groups of interviewed doctors agreed that the ethical principles of autonomy, beneficence, and nonmaleficence should be subordinated to the principle of social justice. This means that in both pre-clinical and clinical triage situations, medical decisions should aim at maximizing the well-being of the collective – whether of wounded or of a society – than at maximizing the well-being of an individual patient. It can be inferred that physicians have a functional and practice-oriented understanding of triage, grounded in a utilitarian approach. It also shows a distinct orientation of ethical perspective of the interviewed civil physicians on military norms of triage. Pre-clinical triage scenarios were evaluated as existential sorting processes, whereas clinical triage scenarios were seen as priority-oriented work. Moreover, the interviewees overwhelmingly agreed that all triage situations necessitate restriction of patient's individual autonomy. In such case, the physician as the ultimate decision-maker decides which therapeutic measures are indicated for individual patients, taking under consideration the availability of resources, the severity of the whole situation, the number of patients requiring help, and, eventually, the individual prospects of success. Furthermore, in a pandemic situation, the interviewees considered the restriction of patient's autonomy regarding treatment discontinuation – ex-post triage – unavoidable. Otherwise, it would lead to a “first-come, first-served” situation, which was medically and ethically inacceptable to a majority of interviewed physicians.

## Data Availability

The raw data supporting the conclusions of this article will be made available by the authors, without undue reservation.
