# Supplementary material for: Ethical aspects of triage in civil and military contexts: a qualitative research of physicians’ attitudes and knowledge regarding ethics in triage scenarios in
Source: Front Med (Lausanne). 2026 Oct 20;13:1938455. doi: 10.3389/fmed.2026.1938455 (PMC13727626; doi:10.3389/fmed.2026.1938455)
Supplement: Supplementary file 1 [file Datasheet1.pdf]

Interview Guide for the Project

Ethical Aspects of Triage in Civilian and Military Context

Personal Experiences

1. In what context have you had experience with the topic of triage?

2. How would you assess your knowledge and skills regarding triage?

Ethical Dilemma

3. How important is it to ensure fairness and equality in the treatment of patients in triage situations?

4. To what extent do you see challenges in implementing equity and equality?

5. In your opinion, are there different ethical principles and objectives in the various scenarios – mass casualty incident, military operation, or pandemic – that would guide your actions in the triage decision-making process?

6. What ethical conflicts and challenges come to mind regarding the triage of patients? In your view, do these depend on the context – mass casualty incident, military operation, or pandemic?

Patient – Physician Relationship

7. In your opinion, how do triage decisions influence the patient-physician relationship?

8. How do you assess patients' self-determination in the context of triage scenarios?

9. In your opinion, how do triage decisions influence a physician's duty of care?

10. How do you evaluate the implementation of the principle of avoidance of harm in triage situations?

11. From an ethical perspective, how do you assess the decision to discontinue treatment that has already begun in favor of another patient?

Approaches to Addressing the COVID-19 Pandemic

12. Are you familiar with the current DIVI guidelines and the current legal situation? If so, how do you assess them?

Feedback and Additional Considerations

13. Thank you very much for participating in this discussion. Finally, are there any other points you would like to highlight or add?
